# An Inquiry into the Therapeutic Value of Sulphate of Magnesia, Oil of Turpentine and Calomel, in Inflammation of Intestinal Mucous Membrane, Especially in Dysentery

**Published:** 1861-09

**Authors:** Wm. Henry Thayer


### CHICAGO MEDICAL JOURNAL.

VOL. IV.] SEPTEMBER, 1861. [NO. 9.

### SELECTED.

### AN INQUIRY INTO THE THERAPEUTIC VALUE OF SULPHATE OF MAGNESIA, OIL OF TURPENTINE AND CALOMEL,

*In Inflammation of the Intestinal Mucous Membrane, especially in Dysentery.*

Read before the Connecticut River Valley Medical Association, July 3, 1861.

By WM. HENRY THAYER, M. D.

In entering upon such an inquiry, which is confined to certain methods of treating acute and chronic dysentery, it is best to define clearly the disease which is intended. Dysentery, then, is an inflammation of the mucous membrane and the submucous areolar tissue of the large intestine. These coats are more or less thickened and reddened—the thickening is sometimes very great, even three or four times their normal thickness, and is the product of inflammatory exudation in and beneath their tissue, which exudation may be thin and serous, or more albuminous and plastic, and may even result in the formation of pus under the mucous membrane. The mucous membrane itself is deeply redened and very soon dotted all over with minute superficial ulcers, round and not more than an eighth of an inch in diameter. It is most common to find the mucous membrane deeply red, swollen and covered with these minute ulcers. The inflammation is usually found to extend from the anus upward more or less, semetimes only through the rectum, at others throughout the descending colon also, at others through the entire large intestine. It is rare, however, to find it above the ileo-cœcal valve. This is, in brief, the anatomy of the disease.

The symptoms are generally proportioned in severity to the extent of the inflammation. In a large part of the cases which are met with in New England, the inflammation is limited to a portion of the large intestine—it is rare to have the whole tract, from ileo-cœcal valve to anus invaded The rectum rarely escapes. When it is not inflamed, we find tenesmus wanting, and the disease is more manageable. The disease begins with general febrile action, some uneasiness in the bowels, loose evacuations, pain, and in the course of twenty-four hours the characteristic discharges, attended with tenesmus, are established. These discharges are generally small, from one drachm to one ounce of mucus, blood and serum, without fæcal matter or odor. When small they are bloody mucus alone, when larger there is often serum with them. As they lie in the vessel they resemble masses of bloody jelly or sometimes broken bits of membrane, and when there is much serum, it is necessary to lift up portions with a stick, when the stringy transparent mucus, more or less stained with blood, is distinctly recognized. The evacuations vary in frequency from the smallest number up to so many that the patient has hardly left the vessel before he is called by another —and the severe pain in the rectum with distressing tenesmus are almost continual. Tenderness over part of the abdomen is a common symptom. It usually marks the extent of the inflammation in the large intestine. Besides the tenderness, however, along the colon, we almost invariably find a spot just below the umbilicus in the median line, which is painful on pressure, evidently not from subjacent inflammation—for the small intestine lies beneath. I have considered the extent of tenderness—indicating the extent of the inflamed portion —a valuable aid in early prognosis, along with the condition of the pulse; but there are cases in which there is no tenderness whatever on pressure over any part of the abdomen, although in all other respects well-marked cases of dysentery.

Dysentery sometimes occurs as a sequel of British typhus, and in these cases it is usually unaccompanied by tenderness. I have seen it so in some of these cases which were fatal after several weeks' illness, and presented after death evidences of intense inflammation of the whole large intestine—thickening, reddening and ulceration.

Tenderness and tenesmus, therefore, may be occasionally wanting; but we always have the characteristic discharges of mucus more or less mingled with blood.

It is not my intention to occupy your time with a complete account of the pathology of dysentery. Since I have said so much, however, I will merely add that vesical tenesmus is frequently a distressing accompaniment of dysentery, and that sometimes we have daily vomiting, but only in very grave cases. Indeed, continued vomiting must induce us to make an unfavorable prognosis.

Such is dysentery, as we see it in the large majority of cases in New England, a painful but not a dangerous disease, if well treated.

There are slight varieties in the character of the evacuations—in the amount of serum, or the occasional presence of bile; I have seen a case in which the blood was entirely wanting, the discharges consisting wholly, from beginning to end, of thick gelatinous transparent mucus, with tenesmus, pain and tenderness, as usual.

When dysentery is epidemic, we may find it having a much graver type than in the sporadic cases which we ordinarily meet with, and resembling more nearly the severe disease of tropical climates and camps. And we may occasionally meet with a sporadic case of violent and fatal character, just as we sometimes see a sporadic case of cholera asphyxia. I remember seeing a young man attacked with dysentery severely, who, before twenty-four hours had passed, was in complete collapse, cold, livid and nearly pulseless, and on the third day was dead. Such a case belongs to the same class with the congestive form of intermittent fever and the malignant form of scarlet fever. It has an element extraneous to the usual character of dysentery, an element which it in common with other acute affections in the rare instances has of the collapse just mentioned.

These cases are exceptional in their treatment, as they are in their nature. The course of medicinal treatment which I shall recommend does not refer to them, but to the cases of dysentery which we most commonly meet with.

There are three indispensables in the treatment of all cases. These are, absolute rest in a recumbent position, constant warmth of the surface, and a limitation of ingesta to a bland liquid diet.

After these comes the medicinal treatment. I will premise by reminding you that the views on the proper treatment of dysentery have varied from time to time for forty or fifty years, partly by the influence of the medical reports of men of the different countries, who have found so marked a variation in the type of the disease in different climates, that they have naturally advocated entirely different methods, which have sometimes been adopted by others with too little regard to varieties of climate and consequent variations of type. The common methods have also been changed from time to time, for sufficient reason found in actual changes in the character of the disease—which has in one epidemic obstinately resisted treatment to which it has been amenable at another time.

Thus the East Indian treatment was adopted in Europe and America—and in a climate so different as that of New England, and a disease so little like East Indian dysentery, blood-letting and salivation were the standard treatment at one time; though apparently with no more reason for it than there would be for that treatment indiscriminately at the present time. Cullen considered the troublesome symptoms in dysentery to be produced by the retention of hardened fæces, and his treatment by purgatives of a mild character has been much followed. I have sometimes seen dysentery begin abruptly with discharges of blood and mucus without any previous evacuation of the bowels. In such a case fæces would probably remain and produce more or less irritation. But in most cases there are several loose, more or less fœcal, evacuations, fully emptying the bowels, before the mucous discharges commence; and cathartics, for the purpose proposed, are needless.

Since the morbid anatomy of dysentery has been more accurately known, a revulsion of opinion has taken place in the medical profession. I well recollect the tone of the late Dr. Enoch Hale, of Boston, an earnest pathologist, on the occasion of the autopsy of a man who died of malignant dysentery at the Massachusetts General Hospital, in 1843, when the intestine was opened and exhibited an unbroken extent of intense inflammation from the ileo-cœcal valve to the anus, and not a particle of fæcal matter to be seen anywhere—when he denounced in no measured terms the plan of applying active purgatives to such a mucous membrane as that, as a proceeding little short of barbarism. So a considerable part of the profession, with the same views, gave up purgatives, and adopted calomel and opium, acetate of lead and opium, or opium alone. Many holding on to the idea of the scybala, give opium and cathartics on alternate days.

Since 1852, I have followed a very different plan in the treatment of dysentery. My patient is put at once on frequently repeated small doses of a saline cathartic. I commonly employ the sulphate of magnesia in doses of one drachm, repeated every four hours. The object is not to get rid of scybala. If I had any reason to suspect their presence, I should give a full cathartic dose of castor oil at once. The object is to relieve the inflammation (or if used at the outset, the congestion) of the mucous membrane by procuring a free serous discharge from its surface. That this effect is produced to the great relief of the symptoms, and usually to the speedy cure of the disease, I have frequent evidence. This will be better understood, if I describe the course. A patient is having frequent dysenteric discharges, with all the other symptoms. He gets one drachm of sulphate of magnesia in concentrated solution, (which is important,) every four hours. The next day I find that his discharges have become large and watery, with little mucus and less blood, are less frequent and less painful. If they have thus improved, I reduce the frequency of the salts, but have it continued every six hours. On the second day my patient has only one or two discharges, watery, and absolutely without blood or mucus. The medicine is ordered once in eight hours, and the next day I find he has had no evacuation at all, and the medicine is omitted. This is the end of the case—for he goes two or three days without any evacuation, if he is careful, and then has a natural discharge. The pain and tenesmus have usually disappeared at the end of the first twenty-four hours, without any opium whatever. This is the history of four cases in five of dysentery when I see them at the beginning. If the case is more advanced, it does not yield so readily, and may require an opiate at night, while the salts are given during the day.

I have sometimes given the salts less frequently, with less favorable results—I have had reason to think that where the symptoms did not yield, it was because the medicine was taken at too long intervals. I prefer to withhold opium, in order that the salts may have their full effect; during the first day, the patient can be soothed and relieved by sinapisms and fomentations, with flannel bands around the body; and after this the pain is relieved.

My own favorable experience of this mode of treatment has been corroborated by others. I am unable to recollect the source from which I first derived the idea, but I have endeavored ever since to disseminate it, and I have had the pleasure within the last six months of seeing the plan advocated in several medical journals.

It is possible that the efficacious use of laxatives is not limited to sulphate of magnesia or to saline substances. I had a theory of their *modus operandi*, derived partly from Golding Bird—that they produced their effect by causing an exosmose of serous fluid from the inflamed mucous membrane, from which arose the necessity of using a concentrated solution. But some experiments by Headland seem to prove that the salts are taken up into the circulation before reaching the inflamed large intestine, and therefore some other explanation of their mode of action must be sought, which shall equally well account for the successful operation of some other cathartics administered in the same manner—that is, in frequently-repeated small doses. I refer particularly to castor oil. By the recommendation of Dr. West, the accomplished senior physician to the Royal Infirmary for Children, London, I have often found great benefit from the use of castor oil in small doses often repeated, in the dysentery of children. I do not know that the sulphate of magnesia would not operate as well with children as it does with adults, but I have never used it. Dr. West's formula for the castor oil emulsion is the following:

R. Ol. Ricini, one drachm; Pulv. Acaciœ, one scruple; Syrupi Simplicis, one drachm; Tinct. Opii, four drops; Aquæ Floræ Aurantii, seven drachms. M. Ft. Mistura. A teaspoonful to be given every four hours.

I have employed it with very happy results in many cases of dysentery of a somewhat chronic character in children. It has the advantage of being not unpalatable—children generally take it without any objection.

Like the epsom salts, castor oil in some way reduces the inflammation of the mucous membrane, and the secretion of bloody mucus rapidly diminishes, simultaneously with the abatement of irritation and pain.

I have spoken thus far of dysentery and its successful treatment, as it is seen in a large majority of the cases in New England. I have had but little experience with malignant dysentery or with grave epidemics, for they are extremely rare in New England. I have no reason to think that the saline treatment would have the slightest influence in malignant dysentery. But in most of the cases that are met with during even a serious epidemic, I have little doubt that, if employed early, it will be found as effectual as it is in the milder form that it takes among us almost every summer and autumn. Its use is not inconsistent with the employment of stimulants, when they are required.

If, nevertheless, there are at any time non-malignant cases which do not yield at all to the use of saline medicines, or which, after a partial improvement, continue in a subacute form, what course promises the greatest and promptest success?

Let me first say that if the dysentery has yielded, but the patient is affected with diarrhœa, astringents and antacids with opium are the most appropriate remedies.

If after two or three days' employment of the saline, the discharges continue to be of bloody mucus, and there is little abatement of the other symptoms, it is time to resort to other expedients. In that case, I presume, no treatment promises better than a mercurial—a small dose of calomel with opium every six hours—under which we may reasonably expect a decided change for the better in the character of the evacuations, before enough has been taken to cause any considerable risk of ptyalism, except where there is an unusual sensitiveness to its effects.

I should say that I have very rarely been obliged to resort to mercurials with adults—for I have found very few cases that did not yield to the sulphate of magnesia. But in the "inflammatory dirrhœa" (West) of children, I have frequently prescribed small and repeated doses of calomel with opium, when other measures failed, and usually with promptly good effect.

From some experience with the oil of turpentine, I am inclined to regard it as a remedy very similar to mercury in its effects upon the intestinal mucous membrane. I have employed it more especially in the dysentery of children. When the castor oil emulsion has been taken several days or a week, and the child, after a partial abatement of the symptoms, has ceased to improve, the addition of a few drops of the oil of turpentine to each dose often has a decided effect upon the symptoms, so that the improvement in the evacuations begins again, and in a few days more they have acquired their healthy character and number. This is the history of favorable cases, which in infants are of course less frequent than in adults.

The value of turpentine in chronic inflammations of the mucous membrane is perhaps sufficiently well known. Professor Wood has brought it into notice as a very useful remedy in an advanced stage of typhoid fever, especially on account of its prompt effect upon the ulcers of Peyer's patches, in promoting their cicatrization.

Three cases of chronic inflammation of the large intestine of moderate extent, mostly in the rectum in two of the cases, have been under my care within a few months past. They were all treated with the oil of turpentine in three daily doses; all began to improve in a few days, and two entirely recovered.

Turpentine is especially useful in dysentery when the first stage of active inflammation has passed, with dysenteric symptoms still remaining, and the patient presenting an appearance of morbid prostration. Exhibiting, in fact, a condition evidently requiring stimulation: feeble pulse, and livid extremities, with tendency to coldness of the surface, and perhaps the tongue dry and the teeth covereed with sordes. Dr. Wood makes the dry black tongue and the sordes the indication for the use of turpentine in fever. He says: "There is a particular state of fever usually attended with much danger, in which we have found this remedy uniformly successful. The condition of things alluded to, is one which occurs in the latter stages of typhoid fevers or lingering remittents, in which the tongue, having begun to throw off its load of fur in patches, had suddenly ceased to clean itself, and becomes dry and brownish. The skin is at the same time dry, the bowels torbid and distended with flatus, and the patient sometimes affected with slight delirium. Under the use of small doses of oil of turpentine, frequently repeated, the tongue becomes moist and again coated, the tympanitic state of the bowels disappears, and the patient goes on to recover as in a favorable case of fever. We are disposed to ascribe the effect to a healthy change produced by the oil in the ulcerated surface of the intestines." (*U. S. Dispensatory*, Art: *Oleum Terebinthinæ.*)

With regard to the use of calomel in dysentery, there appears to be reason for doubt that its efficacy depends upon any direct influence over the functions of the liver, as is very frequently alleged by medical men, or that the necessity for its exhibition rests on any hepatic complication. I shall only argue against these suppositions by the statement of two or three facts of modern observation.

It has been inferred from the green color of the evacuations that is often seen after calomel has been taken, that an excessive flow of bile had taken place into the intestine, under the immediate influence of the mercurial upon the liver. "The green stools," says Pereira, "which sometimes follow the administration of calomel to children, are usually supposed to arise from the action of this medicine on the liver; though Teller thinks it depends on alterations produced in the condition of the blood. The same colored stools are frequently observed when no mercury has been used, and there does not appear to be any just ground for abscribing them to the calomel." It will be remembered that one of the first results of a slight exposure to the cold in an infant, is the passage of green stoods, with griping pain. West says, that in some cases the green discharges probably depend on the action of the acids of the alimentary canal upon the coloring matter of the bile (biliverdine) in the evacuations—which is probably the explanation of these cases of green stools following a chill. When the discharges are greenish in the course of dysentery, Golding Bird's investigations have rendered it probable that it results partly from the presence of altered blood in the evacuations.

Thus we have one fact—that the green stools are very common under various circumstances, unconnected with the use of calomel.

Another fact is, on the authority of Dr. Thudichum, of London, that calomel, whether it purges or not, does not increase the quantity of bile excreted, but on the contrary it diminishes it. If the liver is relieved by the use of calomel, it is through its effect upon the intestines and the portal circulation, just as it would be relieved by other cathartics. This is proved by the experiments of H. Nasse, Kolliker, and H. Muller. The green color of the stools which follow the use of calomel is really due to sub-sulphide of mercury, just as the black color of stools following the use of preparations of iron, is due to sub-sulphide of iron. (*Thudichum.*)

We have reason, then, to know that calomel does not produce that increase in the biliary secretion and discharge, which many have considered indispensable to the relief of various cases of intestinal disturbance. And we may fairly infer that whatever advantage is derived from the use of calomel, as of turpentine in dysentery, is due to its direct effect upon the capillaries of the intestine, as a special stimulant.

Whether turpentine might not be employed with as great advantage as calomel in cases that seem to require either of them, my own experience will not allow me to say. I have seldom used calomel since I began to treat dysentery with sulphate of magnesia, nine years ago. Calomel would be unsafe in a very advanced stage of the disease, when there was any pus in the discharges, and the vital force was low—while these are the very conditions in which the use of turpentine, a nervous stimulant, would be especially appropriate. (*Berkshire Medical Journal.*)

